# A study of logon-evoked middle latency responses in female subjects with normal hearing

**DOI:** 10.1016/S1808-8694(15)30073-2

**Published:** 2015-10-19

**Authors:** Erika Maria Fukushima, Ney Penteado de Castro

**Affiliations:** a Physician, graduate student (master’s degree), Santa Casa Medical School, Sao Paulo; b Doctor in Otorhinolaryngology, adjunct professor in the Otorhinolaryngology Department of the Santa Casa Medical School, Sao Paulo

**Keywords:** normal hearing, mlr, logon, auditory evoked potential

## Abstract

The middle latency response (MLR) to an acoustic stimulus occurs between 10 and 80 ms. The waveform is characterized by a series of peaks and troughs labeled N0, P0, Na, Pa, Nb and Pb. Certain acoustic stimuli may excite specific cochlear areas in contrast with clicks, that activate the cochlea between 1000 and 4000 Hz. The logon stimulus activates segmentar areas of the cochlea and has advantages over clicks when assessing low frequency areas of the cochlea (below 1 kHz). **Aim:** The aim of this paper was to study the MLR electrophysiologic response when activated by logon stimuli at 500, 1000 and 2000 Hz. **Method-** a prospective and descriptive study. 14 female volunteers had normal otology and conventional audiology results. The stimulus was monoaural and ipsilateral (Cz/A1-2). **Results-** the NaPa complex was readily identified compared to other complexes and was present in 100% of the tests done at 2000 Hz, and in 96.4% of the tests done at 500 and 1000 Hz. **Conclusion-**the logon stimulus at 500, 1000 and 2000 Hz elicits MLRs; the NaPa complex was the most frequent event and the 2000 Hz frequency elicited more responses than other frequencies.

## INTRODUCTION

Hearing is part of a specialized communication system that includes much more than only peripheral sensitivity. This complex sense allows individuals to identify, locate and process sounds, enabling persons to monitor danger signs, to appreciate music and to understand speech.

Integrity of the whole path traversed by a sound stimulus, from the external ear to the central auditory pathways, is required for this sense to be fully used. The neuronal nuclei located in the bulbus, pons, mesencephalon and diencephalon process sound specifically and hierarchically until it reaches the auditory cortex.

The assessment of function of this pathway may be done using auditory evoked potentials, which comprise the technical ability to capture electrical events that are evoked along the auditory system. When these tests are used jointly, the result is a global, detailed and distinct analysis of the various instances of the auditory pathway.^1^

Auditory evoked potentials may be classified according to their latency; middle latency potentials or responses (MLRs) are waves obtained 10 to 80ms after a sound stimulus.^2^, ^3^, ^4^, ^5^ These waves are also known as auditory middle latency responses (AMLR), auditory middle responses (AMR), middle latency auditory evoked potentials (MLAEPs), and middle latency evoked responses (MLER). MLRs are characterized by a polyphasic potential of neuronal origin that occurs sequentially to the auditory brainstem response or evoked potential (ABR). Evoked waves are named according to their latency and amplitude phase, as follows: N0, P0, Na, Pa, Nb and Pb (this last wave is also known as P1 or P50).^6^, ^7^, ^8^

Most of the published papers on this theme refer to these components from the Na wave onwards, as sonomotor responses are purely myogenic in origin and may temporally overlap lower latency waves.^8^ The myogenic response is characterized by a negative-positive biphasic potential with latency times between 12 and 15ms and 18 and 25ms after a sound stimulus; there may also be positive deflection. The myogenic response is usually observed at the beginning of a session when the subject is not sufficiently relaxed, or when a strong stimulus is used; it may also occur as a result of electrode placement, particularly when placed over the mastoid process. The myogenic response occurs mainly by contraction of the posterior auricular muscle, and to a lesser degree by contraction of other muscles of the scalp, such as the temporal and frontal muscles.^1^, ^2^, ^9^, ^10^, ^28^

The origin of MLR wave generators is uncertain. Deflections mirror the auditory pathway segment located between the inferior colliculus and the medial geniculate body to the temporal lobe, namely the medial portion of Heschl’s gyrus (subcortical or cortical regions).^11^, ^12^

Early auditory potentials and ABRs are neuronal potentials that require synchronous events to take place; the most frequently used stimulus is a click between 1 and 4 kHz.3 MLRs are post-synaptic potentials^9^ that originate in dendrites and that may be elicited by clicks^13^, ^14^, ^15^, ^16^ or by tone pips,^10^, ^15^, ^17^, ^18^ tone bursts^3^, ^4^, ^19^ or logon.^1^ This opens the possibility of assessing specific frequencies using MLRs, as central auditory connections preserve the cochlear tonotopy.

Click-obtained MLRs topographically supplement ABR data. Clicks are also the most frequently used stimulus in neurological assessments; clicks stimulate the cochlea nonspecifically between 1kHz and 4kHz. When using acoustic stimuli that allow a choice of frequency, such as the logon, the test becomes especially useful. It is then possible to evaluate low frequency sound thresholds,1,3 making it possible for this test to be done in patients with severe-deep hearing loss at high frequencies, which is not possible with tests that use clicks.

Thorough studies are needed both in normal subjects (to test reproducibility and normalization) and in subjects with the disease to be investigated (test accuracy) for a test to be recognized as clinically useful. Although MLRs were described many years ago, studies on the nature of this response are still done to increase knowledge about its features, caveats and its appropriate use. Improved use of MLRs as a clinical tool requires understanding the stimulus-response relation for a given stimulus frequency.

Logon is a short stimulus similar to a Gaussian curve (sine wave) that makes it possible to assess MLRs individually at 250 to 8.000 Hz.20 Based on this feature, this paper aimed to investigate the feasibility of using logon stimulus to obtain MLRs, given that logon has not been well studied particularly in MLRs and in human beings.^1^, ^21^, ^22^

## OBJECTIVE

To investigate the electrophysiological response of logon stimulated MLRs at 500, 1.000 and 2.000 Hz in young normal hearing female healthy adults.

## SERIES AND METHODS

The sample included speech therapy students and audiology trainees from the Otorhinolaryngology Unit of the Santa Casa Medical School, Sao Paulo, who agreed to participate.

All of the volunteers signed a free informed consent form before data collection, according to the regulations of the Research Ethics Committee of the Sisterhood of the Santa Casa de Misericordia, Sao Paulo, which approved the study on 26 January 2005 (project number 016/05).

Inclusion criteria were as follows:

• An audiological assessment based on the following standards of normality:
  - pure tone audiometry showing thresholds below or equal to 25 dB NA at 250Hz to 8kHz.
  - a speech recognition rate over 88%
  - a type A tympanometric curve
  - presence of contralateral stapedian reflexes at 500Hz to 4kHz.

Exclusion criteria were as follows:

• the presence of neurological diseases
• users of medication acting on the central nervous system

After clinical history-taking and otoscopy, an audiological assessment was done in the speech therapy unit of the otorhinolaryngology discipline at the abovementioned institution. An acoustically treated booth with faradic damping was used. MLRs were obtained with an Amplaid MK^22^ device and TDH-39 earphones. Subjects were placed in lateral decubitus during the test and lighting was subdued to obtain maximum relaxation of neck and face muscles. The skin where electrodes were to be placed was cleaned with an abrasive paste and electrolytic gel was used between the skin and the electrodes. Electrodes were placed in the Cz/A1-2 derivation (vertex/right-left ear lobules); the ground electrode was placed on Fpz (frontal region) according to the 10-20 system for electrode placement. Electrodes were fixed with MicroporeR.

Impedance between electrodes was kept below 5 kΩ and background noise was maintained below 20µV. High-pass and low-pass filters were set at 10 Hz and 200 Hz. The window of analysis was 100ms.

The peak SPL and logon stimulus was 100 dB at 500Hz, 1kHz and 2kHz, with a 7/s stimulus rate, a total of 1024 stimuli for each frequency analyzed. Stimulus was monaural and ipsilateral to the potential-recording derivation. The initial ear and the sequence of tested frequencies were chosen randomly for each subject.

The test was repeated at least twice; tracings were compared with each other and the examiner confirmed the reproducibility of potentials. Only then each component was studied and latency times in milliseconds were checked and tabulated.

### Statistical analysis

The paired t test and the Wilcoxon test were used to compare the latency time between right and left ears. Analysis was based on the statistical software SPSS 13.0 for Windows, and the significance level was 5%.

A 95% confidence interval was calculated for the means of latency times for each wave. Normality was tested using the chi-square test.

## RESULTS

The sample included 14 female white volunteers aged between 17 and 27 years (mean age - 21 years, standard deviation - 2.67). Testing took about 45 minutes and most of the subjects slept at some point during the test. One subjects presented myogenic interference, which entailed repeating the test a number of times.

The easiest waves to detect were Na and Pa, which became the reference for other components. Latency times were tabulated and treated statistically.

Latency times were compared using the paired t and the Wilcoxon tests to analyze right and left ear responses. No statistically significant difference was found between right and left ears, so it became possible to analyze the data jointly (28 tests at each frequency).

The Na-Pa complex was present in 100% of tests at 2000 Hz, and in 96.4% of tests at 500 Hz and at 1000 Hz. Presence of deflection in decreasing order were Nb, Pb, P0 and N0, except for Pb at 500 Hz that showed a higher number of responses than Nb at this frequency. The N0 P0 complex was not identified at 500 Hz (Table 1).

**Table 1:** Number of valid responses per wave at 500, 1000 and 2000 Hz, and the percentages.

	500 Hz		1000 Hz		2000 Hz	
waves	number of cases	%	number of cases	%	number of cases	%
N0	0	0,0	5	17,9	5	17,9
P0	0	0,0	5	17,9	6	21,4
Na	27	96,4	27	96,4	28	100,0
Pa	27	96,4	27	96,4	28	100,0
Nb	13	46,4	15	53,6	15	53,6
Pb	15	53,6	9	32,1	10	35,7

**Figure 1:**
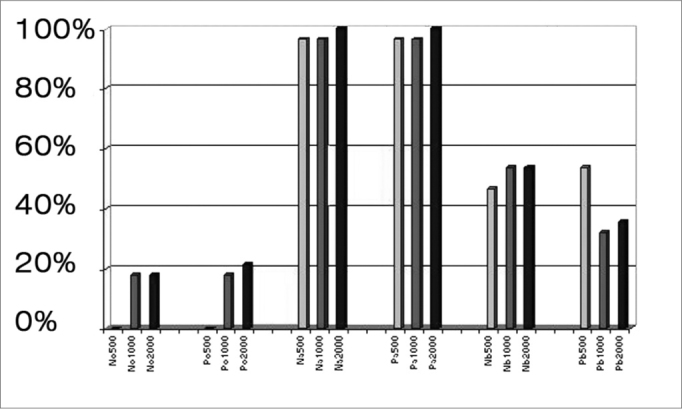
Percentage of N0, P0, Na, Pa, Nb, and Pb waves at 500, 1000, and 2000 Hz.

At 2000 Hz there was a higher percentage of P0, Na and Pa responses compared to responses at other frequencies. N0 and Nb had the same number of responses at 1000 and 2000 Hz and a lower number of responses at 500 Hz. Pb responses decreased with frequency at 500, 2000 and 1000 Hz (Figure 1).

Table 2 shows summarized measures for each wave at 500, 1000 and 2000 Hz. Waves with the lowest latency time variability were Pa at all frequencies and Nb at 500 and 1000 Hz. The component with the highest coefficient of variation was Pb at all the tested frequencies.

**Table 2:** Distribution of wave results per frequency, with minimum and maximum values, the mean, the standard deviation, the coefficient of variation and the median (in milliseconds).

wave	minimum	maximum	mean	standard deviation	c.v. (%)	median
Na500	17,2	26,0	21,75	2,1	9,7	22,0
Pa500	24,8	33,6	30,34	1,9	6,4	30,4
Nb500	34,4	42,0	38,74	2,0	5,2	38,8
Pb500	41,2	67,2	47,25	5,9	12,6	45,6
No1000	11,6	15,6	12,80	1,6	12,7	12,0
Po1000	14,8	18,8	16,24	1,6	9,9	15,6
Na1000	16,8	24,8	20,00	2,1	10,3	19,6
Pa1000	26,8	33,6	30,09	2,1	6,9	30,0
Nb1000	34,4	42,8	37,92	2,5	6,6	37,2
Pb1000	40,0	62,0	47,38	6,7	14,2	45,2
No2000	10,0	12,8	11,44	1,3	11,2	10,8
Po2000	14,4	16,8	15,53	1,0	6,2	15,8
Na2000	16,4	23,2	19,06	1,6	8,4	18,8
Pa2000	26,0	34,4	29,34	2,1	7,3	28,8
Nb2000	34,0	53,6	39,41	5,3	13,5	37,6
Pb2000	42,4	62,0	47,12	7,4	15,6	44,0

c.v. - coefficient of variation

The 95% confidence interval for the mean was calculated when the hypothesis of normality was not rejected in the chi-square test (p >0.05). See Table 3.

**Table 3:** Confidence intervals of the latency time means for waves No, Po, Na, Pa, Nb and Pb at 500, 1000 and 2000 Hz.

Wave	500 Hz	1000 Hz	2000 Hz
N0		10,8 a 14,8	9,8 a 13,0
P0		14,2 a 18,2	14,5 a 16,5
Na	20,9 a 22,6	19,2 a 20,8	18,4 a 19,7
Pa	29,6 a 31,1	29,3 a 30,9	28,5 a 30,2
Nb	37,5 a 39,9	36,5 a 39,3	36,5 a 42,4
Pb	43,9 a 50,5	42,2 a 52,5	41,8 a 52,4

## DISCUSSION

MLRs were described for the first time in 1958 by Geisler et al.^23^, but only came to attention for clinical use in 1980 due to the technological development of signal averagers, which enabled potentials to be detected with increased reproducibility and sensitivity. At present there is no universal consensus on a MLR test protocol, resulting in difficulties for generating absolute latencies for MLR components. It is generally assumed that these latencies occur between 10 and 80ms after a sound stimulus.^2^, ^3^, ^4^, ^5^

Myogenic contamination initially caused much debate about MLR generators,^24^, ^25^ which led to many studies until the neurogenic origin of these potentials was demonstrated.^26^, ^28^ It is currently known that myogenic responses of the scalp and neck muscles may be elicited by strong acoustic stimuli and/or by difficulties in relaxing the muscles during the test.^1^, ^9^, ^10^, ^27^ Good technique for MLR testing consist of avoiding these artifact-generating situations that can compromise test results. Placement of the reference electrode on the ear lobule rather than the mastoid process also helps to avoid myogenic contamination. In our study there was interference from myogenic potentials in one test, which affected the reproducibility of elicited responses; the test had to be repeated a number of times.

Prevalent waves were Na and Pa at all frequencies, varying from 96.4% present (at 500 and 1000 Hz) to 100% present (at 2000 Hz). Other complexes showed decreased reproducibility, varying from zero to 17.9% for N0P0 and from 32.1 to 53.6% for NbPb (Table 1), which is similar to results in the literature.1,9 The N0P0 complex may be overlapped by myogenic activity or the sonomotor response. There are authors, therefore, that recommend the analysis of MLR-generated events from Na onwards.^8^, ^10^ Na and Pa waves are more reproducible responses and consequently are the most analyzed waves; they are considered reliable components for the various stimuli that are tested.^1^, ^2^, ^29^, ^30^ The NbPb complex is the last of the MLR, and its reproducibility and latency is variable, which affects the analysis.^15^, ^16^

Our data revealed no difference in latency time variability at the frequencies that were tested (Tab 2). Thornton et al.^19^ observed decreased latency with increased frequency. The 2000 Hz frequency elicited the highest number of responses except for Pb, which was mostly present at 500 Hz (Table 1).

MLR wave amplitude and latency are influenced by the intensity and the stimulus rate. Amplitude is directly proportional to the intensity of the sound stimulus.^17^, ^23^ Intense sound stimuli, however, elicit significant myogenic activity and were avoided in this analysis. Amplitude is inversely proportional to the stimulus rate; there are reports showing that stimulus rates over 10/sec significantly compromise MLR amplitude.^10^, ^23^, ^27^ Pb is the most sensitive wave to the stimulus rate and is elicited ideally at 1/sec.^11^, ^31^ In this paper the stimulus rate was defined as 7/sec, which elicited MLR waves and shortened the test time.

Waves Na, Pa, Nb and Pb are more robust and therefore easier to detect, however amplitude is not a reliable parameter due to significant intra- and intersubject variability.^5^, ^15^ On the other hand, peak latency is relatively stable,6 and is used in every study of MLR.

Waves with the lowest variation coefficient, those that showed the lowest latency variability, were Pa at all frequencies, Nb at 500 and 1000 Hz, and P0 at 2000 Hz. N0 and Pb latencies had the highest coefficient of variation among all frequencies except for Nb at 2000 Hz (Table 2). According to Schneider,1 the highest coefficients of variation were found in peripheral components (P0, Nb and Pb) and the lowest coefficients were found in central components(Na and Pa).

There is no consensus in the literature on the type of stimulus used for eliciting MLR. A significant number of papers on MLR use clicks as a qualitative test for locating auditory deficiency, particularly in neurological conditions. Examples of this use include patients with multiple sclerosis, cerebral tumors, epilepsy, traumatic brain injury and learning disabilities.^8^ A narrow band stimulus capable of stimulating specific cochlear regions could be used clinically^19^ to quantitatively assess hearing loss. In other words, establishing electrophysiological thresholds at a specific frequency would truly be an electrophysiological pure tone audiometry. This type of MLR would be ideal to assess subject with severe hearing loss at high frequencies,1 as it would be possible to estimate the electrophysiological auditory threshold using stimuli at various frequencies. Stimuli that elicit MLR and make assessments by specific frequencies possible are tone bursts,^3^, ^4^, ^19^ tone pips^10^, ^15^, ^17^, ^18^ and logon.^1^ We employed logon in three frequency octaves, 500, 1000 and 2000 Hz.

MLR latency increases when the stimulus intensity decreases;^1^ Na and Pa waves may be obtained^11^ even when the stimulus intensity is close to the subject’s psychoacoustic threshold. A few authors have reported that under these conditions these waves are more easily detected than the V wave,^13^, ^29^ although other have contented that ABR would be superior to identify auditory sensitivity compared to MLR.9 Still other authors believe it is important to use both tests for a more encompassing assessment of auditory pathways.^1^

In our paper ear laterality was not statistically significant, a finding that concurs with Tucker et al.’s7 results, where no Pa latency differences were uncovered. Matas et al.,^16^ however, noted differences in Pa, Nb and Pb, which were more prolonged to the right.

One should bear in mind that, when using the confidence interval for mean latency times, this study investigated only young female subjects. Although most authors have not found significant gender differences in MLR8-9 a few papers have reported decreased Pa latency and increased Pa amplitude in women.^31^

Further studies that include men and a wider age range are required for increased population representativeness.

## CONCLUSION

Our conclusion about MLRs in a sample of normal hearing female subjects was as follows:

- MLRs may be elicited by logon stimulus at 500, 1000 and 2000 Hz;
- the Na-Pa complex was the most reproducible event of the MLRs;
- logon at 2000 Hz elicited a higher response number compared to other frequencies.
